# Hospitals' Association Conference

**Published:** 1911-06-10

**Authors:** E. C. Smith

**Affiliations:** Secretary. North Ormesby Hospital, Middlesbrough


### THE BRITISH HOSPITALS' ASSOCIATION.

### HOSPITALS' ASSOCIATION CONFERENCE.

Sir,—The question of the State Insurance Bill as it affects hospitals is neither medical nor surgical but purely financial; consequently I was rather surprised at the number of medical men present at the meeting of the British Hospitals Association on Friday last. I think it would have been very much more interesting if the views of more hospital secretaries could have been obtained, but from what was said by the different speakers everyone seemed to agree that hospitals would suffer by the working of the Bill as at present constituted, and it is only in minor details that opinions vary. The appointment of a committee to interview Mr. Lloyd George, which is undoubtedly a step in the right direction, must be most gratifying to all interested in hospitals, and it is to be hoped it will have the desired result.

The gentlemen, whose names were read out by the chairman, with the exception of about three, belong to hospitals in London or in the immediate vicinity (if I remember rightly), consequently they are not a very representative body.

Not a single hospital in Wales, in Newcastle, in Cleveland or in the north-east of England is directly represented. Newcastle receives more subscriptions from workpeople than any other hospital, and practically every hospital throughout Cleveland derives nearly the whole of its income from employers or employees. If, as appears to be the opinion of many people, hospitals deriving the greater portion of their incomes from these sources are likely to be most affected by the Bill, the hospitals in the Middlesbrough portion of Cleveland will be the greatest sufferers, as in very few works do the employees subscribe less than 1½d. per man per week, whilst in a great number of works the amount subscribed is 2d. per man per week.

I think it would have been much better to have made that committee as representative of the different *districts* (I do not of course mean every hospital or even town) as possible, as however the members of the committee were given power to add to their number they may possibly take steps to make themselves a more representative body.—Yours faithfully,

E. C. Smith, Secretary.

North Ormesby Hospital,
Middlesbrough, June 5.

